# Psoriasis to Psoriatic Arthritis: The Application of Proteomics Technologies

**DOI:** 10.3389/fmed.2021.681172

**Published:** 2021-11-16

**Authors:** Fei Qi, Yaqi Tan, Amin Yao, Xutong Yang, Yanling He

**Affiliations:** Department of Dermatology, Capital Medical University Affiliated Beijing Chaoyang Hospital, Beijing, China

**Keywords:** psoriasis, psoriatic disease, psoriatic arthritis, proteomics, biomarkers

## Abstract

Psoriatic disease (PsD) is a spectrum of diseases that affect both skin [cutaneous psoriasis (PsC)] and musculoskeletal features [psoriatic arthritis (PsA)]. A considerable number of patients with PsC have asymptomatic synovio-entheseal inflammations, and approximately one-third of those eventually progress to PsA with an enigmatic mechanism. Published studies have shown that early interventions to the very early-stage PsA would effectively prevent substantial bone destructions or deformities, suggesting an unmet goal for exploring early PsA biomarkers. The emergence of proteomics technologies brings a complete view of all involved proteins in PsA transitions, offers a unique chance to map all potential peptides, and allows a direct head-to-head comparison of interaction pathways in PsC and PsA. This review summarized the latest development of proteomics technologies, highlighted its application in PsA biomarker discovery, and discussed the possible clinical detectable PsA risk factors in patients with PsC.

## Introduction

Psoriatic disease (PsD), as an umbrella term, describes a systemic inflammatory disease that predominantly affects the skin [cutaneous psoriasis (PsC)] and musculoskeletal features [psoriatic arthritis (PsA)], with ~125 million patients worldwide (1, 2). The concept of PsD indicates the realization of the common inflammatory and metabolic pathways working on the skin and synovium (3). Although it is still controversial whether PsC and PsA shared the same immunological factors or belonged to the same spectrum of diseases, studies from genetic and proteomics confirmed the overlap between PsC and PsA (4–8).

Psoriatic arthritis is characterized by multiple joints stiffness, pain, and swelling with insidious onset (1, 9). Poor prognosis with debilitating joint destruction brings a tremendously negative impact on the life quality of all patients (10). It affects one in five people who have a psoriasis diagnosis, while only 15% of PsA cases get cutaneous lesions after arthritis onset (11, 12). After the initiation of psoriasis, the prevalence of PsA grows over time, hitting 20% after 30 years (13, 14). It is significant to identify patients who are at risk for PsA and enable targeting therapies to prevent and intercept the joint involvement at a very early stage of the psoriatic arthropathy (15, 16). A 6-month delay in joint destruction detection is linked to a significantly lower treatment response (17).

Psoriatic arthritis was strongly associated with nail, scalp, skinfold, elbow/knee involvement, the severity, early onset age, and total disease time of the cutaneous presentation (18–20). Symptoms like arthralgia in female psoriasis patients indicated a high chance of developing PsA (21). Although not all PsO patients with joints pain have PsA, a longitudinal study confirmed that compared with psoriatic patients without joint complaints (PsO), those with arthralgia (PsOAr) were more likely to develop PsA in the subsequent follow-up period (22).

Psoriatic arthropathy, an early stage of joint involvement that may not fulfill the PsA diagnostic criteria, is more common than PsA in PsO patients (23). For those with asymptomatic joint abnormalities, early synovio-entheseal inflammation or bone erosion can be detected by imaging features like ultrasonography or MRI (24, 25). However, with these predictors, it is still hard to foresee the possibility of the transition to PsA (26). Unlike rheumatoid arthritis (RA), the absence of serum diagnostic biomarkers impedes the identification of very early PsA from PsC patients (8, 9, 27).

“Omic” technologies have achieved enormous progress in their development and application over the past decades, which provided an unprecedented opportunity to decipher the entire genes (genomics), mRNA (transcriptomics), proteins (proteomics), and metabolites (metabolomics) of a specific biological sample (28, 29). Notably, advances in proteomics have made it possible for the head-to-head comparisons of potential biomarkers in the heterogeneity of PsD (8, 30). The present article reviewed the latest development of proteomics technologies, summarized its application in PsA biomarker discovery, and discussed the possible clinical detectable PsA risk factors in PsC patients.

## RECent Developments in Proteomics Technologies

Proteome, as the ultimate goal for biomarker discovery, is the analysis of the whole protein materials of a disease or a biological sample, which offers possibilities to track the changes in protein expression under different conditions (31, 32). Present proteomic technologies could be addressed either as system-wide and unbiased tools such as antibody-based assay, aptamer-based assay, and mass spectrometry (MS) or a highly sensitive targeted immunoassay, such as the proximity extension assay (PEA) (33–35).

Mass spectrometry is a powerful and flexible instrument for characterizing proteins in their entirety (36–38). Of note, the introduction of high-throughput and high sensitivity protein identification and quantification methods to the single-cell proteomics and multi omics technologies help identify the candidate biomarkers in a protein-centric molecular way (29, 39–41). Ample studies have shown that the protein expression profile in the serum of patients with PsC or PsA can be illustrated *via* multiple MS approaches, including data-dependent methods (such as label-based, label-free, MuDPIT, and shotgun proteomics) and targeted data-independent approaches (such as SWATH and MSE, multiple reaction monitoring, phospho-, and ubiquitinoylation-targeted proteomics) (35, 42). Furthermore, an emerging concept of “proteogenomics” produced fused the insights of proteomic and genomic, in which genomic events, such as SNPs, mutations, insertions, deletions, and substitutions and be detected with a better understanding of its effects at the protein level (43–45). With the help of a series of peptide-to-spectra matches (PSM) by assigning fragment ion mass spectra to peptide sequences, which is similar to proteomics, proteogenomics query the search engines with a customized protein FASTA, which contain both genomes- and protein-modified sequence (46). More recently, an integrated proteomics pipeline (IPP) was established to combine a variety of search engines to improve the sensitivity of novel peptide identifications with a novel “cascade search” method, which maximizes the accuracy and reliability of new candidate biomarker discovery. The current proteogenomics application mainly focuses on precision oncology, which assists in differentiating the subtypes and relevant pathways of tumors (47–53). Although no studies have shown its application on rheumatic diseases, proteogenomics is now the primary suggestion for PsC/PsA biomarker discovery (2, 30).

Proximity extension assay is a novel technology with up to 96-plex immune assays invented by Olink Proteomics (Uppsala, Sweden), which consolidates quantitative real-time PCR (54, 55). It was based on a dual recognition of selected antibodies with which biomarker-specific DNA “barcodes” oligonucleotides were labeled. The unique DNA will be merged by high-throughput relative quantification microfluidic qPCR for up to 1,161 human proteins in the plasma (54, 55). Compared with LC-MS/MS, PEA covers a broader dynamic range with higher sensitivity, which provides sensitive and specific detection of low-abundant proteins in human blood and other body fluid samples (55–58). Moreover, PEA also tends to be less influenced by multiplex ELISA technical problems, such as antibody crossreactivity and interassay variability (59). PEA has been widely applied in non-clinical biomedical research to decipher minute protein concentrations in minute sample volumes. In contrast, current studies have seen more applications of PEA in exploring both diagnostic markers and inflammation key components (60, 61).

## PROTEOmics in Potential Biomarker Discovery of PsA Transition

**Figure 1:**
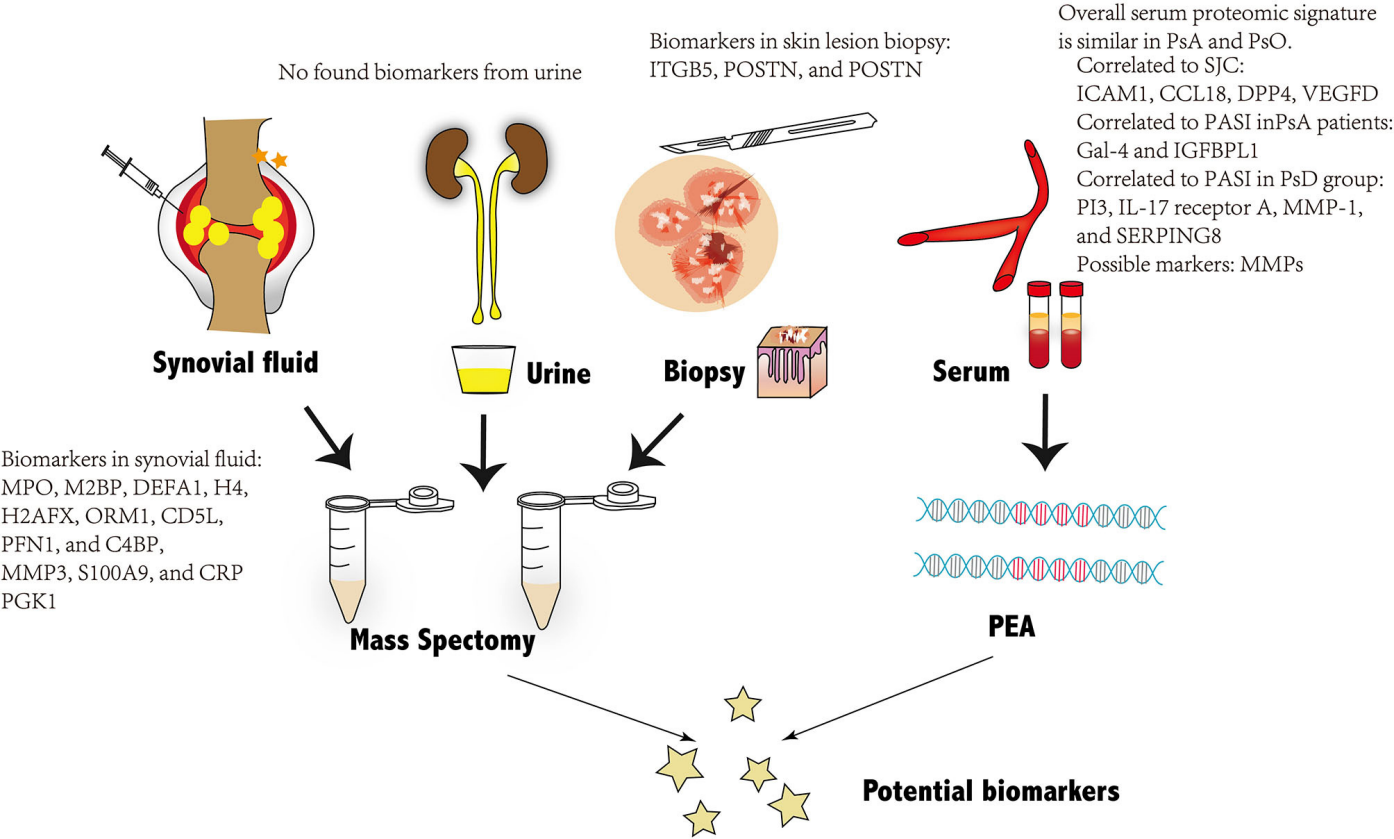
Overview of the potential biomarkers of PsA found through proteomic technologies from four different kinds of biological samples: Biomarkers from synovial fluid and skin tissues are presented in the figure. No possible biomarkers are found from urine and serum. PsA, psoriatic arthritis; PEA, proximity extension assay.

Identifying early asymptomatic PsA in patients with PsO has been recognized as a historically complex issue with no exact serum diagnostic biomarkers used in daily clinical practice (8). Proteomics is extensively adopted in biomarker exploration. The emergence of proteomic technologies allows deciphering the changes in protein expression under diseased conditions. The following session of this work will review the detected possible predictors that may indicate early preclinical and subclinical PsA under the novel proteomics technologies (62) (Figure 1).

### Proteomics in Peripheral Blood

Plasma and serum are extensively applied for proteomics-based biomarker discovery (63). Plenty of studies highlighted both PsA diagnostic and prognostic biomarkers with the help of MS proteomics technology (64). Serum proteome can also be obtained by PEA, an emerging technology previously explored in immune-mediated diseases of the skin, such as atopic dermatitis (65, 66). In a head-to-head comparison of serum biomarkers between PsC and PsA, Leijten et al. chose a high-throughput serum biomarker platform (Olink) to evaluate the concentrations of 951 serum proteins in both patients with PsA and PsC. Although no biomarkers with a significant difference were found between PsC and PsA, PASI scores were found most strongly correlated to the proteins PI3, IL-17 receptor A, MMP-1, and SERPING8, when patients with PsA and PsO belonged to one group. When analyzing PsA patients as one group separately, PASI score was found correlated to Gal-4 and IGFBPL1. Four proteins including Intercellular adhesion molecule-1 (ICAM1), CC chemokine ligand 18 (CCL18), Dipeptidyl-peptidase 4 (DPP4), Vascular endothelial growth factor D (VEGFD), were found correlate to arthritis activity evaluated by swollen joint count (SJC), among which ICAM-1 and CCL18 were reported relevant to synovial tissue in rheumatoid arthritis activity. The swollen joint count (SJC) was identified, among which ICAM-1 and CCL18 were reported relevant to synovial tissue in RA, whereas VEGFD was proposed to participate in the pathogenesis of arthritis. DPP4 was only found to be related to type 2 diabetes mellitus rather than in arthritis development (8, 67–71).

It was found that there were 20 dysregulated proteins, which specially existed in the serum of patients with PsA, which showed at the normal range in the PsO group when compared with the health control (8). Though the published research suggested, it is difficult to find a simple diagnostic protein from the serum to discriminate patients with PsA from patients with PsO, there is still a scarcity of serum proteome with PEA technology, and the mentioned study was completed with a small number of samples. Besides the 11 selected platforms encompassing only inflammatory proteins, more proteins reflected bone turnover and tissue biological changes, such as matrix metalloproteinase (MMPs) (72).

Although human plasma is believed to be a feasible and less invasive source with a rich proteome, potential biomarkers secreted by the targeted tissues may be diluted in the blood with an undetectable concentration by current MS methods (73). In addition, many coexisting factors in the peripheral blood may interfere with the candidate soluble potential proteins. Thus, other biological samples, such as synovial fluid (SF) and skin, have drawn more interest to be analyzed (74). Besides, some authorities recommended a more specific method to finding serum markers after the proteomics of inflamed synovial biomarkers (75).

### Proteomics in Synovial Samples

Synovium is the primary affected site in most inflammatory arthritis (74). Many pathological modifications in inflamed synovial tissue are mirrored in the SF, which was more easily accessible and widely studied (76). SF is a versatile source for proteins from the synovial membrane, cartilage, and plasma, depicting the pathophysiological issues that cause arthritis (77). A previously performed label-free MS quantitation of SF proteomics identified and verified 12 candidate PsA markers, including MPO, M2BP, DEFA1, H4, H2AFX, ORM1, CD5L, PFN1, and C4BP, as well as the top three upregulated proteins: MMP3, S100A9, and CRP (78). In another age-matched study, 10 SF samples from patients with PsA who were examined by using liquid chromatography-tandem MS quantitation revealed that Periostin (POSTN) and phosphoglycerate kinase 1 (PGK1) were upregulated with folded ratio compared with healthy controls (79). Although both studies showed a promising direction in SF proteome, no available data compared SF biomarkers between PsA and PsC samples.

The acquisition of SF is more feasible than synovial tissue, but it is undeniable that SF sometimes provides only indirect biomarkers (80). In the study of RA, the analysis of synovial tissue samples offered great insights into both epigenetic and proteomic changes in patients with very early-stage RA. Therefore, synovial tissue might also be helpful and become a more precise target source in investigating PsA (74, 81).

### Proteomics in Skin Lesion Biopsy

Skin manifestations, which include psoriasis Vulgaris or plaque psoriasis, were strongly associated with PsA (82). One hypothetical model for PsA transition was a systematic expansion of inflammation from the skin to synovio-entheseal tissues (62, 83). Factors that caused cutaneous diseases in the skin were released to promote a systemic dysregulated immune-mediated response and to develop musculoskeletal lesions after a second hit, such as trauma, infection, etc. (84, 85). Hence, it is of great need to explore the skin proteome in patients with PsA and PsC. Label-free quantitation of skin proteins verified 47 different peptides between samples in the two groups. After validation in serum by ELISA, integrin β5 (ITGB5), a group of transmembrane receptors function on cell adhesion, increased significantly in the PsA group when compared with the PsC group. Besides POSTN, a secreted extracellular matrix protein originally derived from the osteoblasts, was believed as a potential serum biomarker with a slightly higher concentration in PsA patients than in PsC patients (86). Another latest research using isobaric tags for relative and absolute quantitation (iTRAQ), a labeled MS technology, found 2-5-oligoadenylate synthase levels in both serum and psoriatic epidermis that were positively correlated with the severity of psoriasis through PASI and BSA (87, 88). As some data suggest, severe psoriasis can account for another cutaneous feature with a higher risk and prevalence of psoriatic arthropathy. The plasma membrane ATPase (derived from the OSA2 gene) might become another possible predictor for early joint inflammations in psoriatic patients (89, 90). Although these results are promising, limitations such as small sample numbers and the absence of further repetitive investigations in skin proteome impede the uncover of candidate PsA biomarkers, as well as the understanding of the underlying mechanism. There is no published research involving synovial tissue proteome in patients with PsA or PsC. Farnebo et al. performed MS analysis on a rabbit tendon injury model to compare protein expression in intrasynovial tendon grafts and extra synovial tendon grafts, which offered a possible substitute for the hard-to-access human samples (91).

### Proteomics in Urine

Urine is another excellent source for both systemic and renal inflammatory biomarker exploration for its non-invasive sample collection approach as well as the low dynamic analytes range (92). Most proteins identified in urine are filtered from the plasma or generated by inflammatory renal cells, contributing to a relatively small number of proteins appearing in the urine in patients with normal kidney function (93). Meanwhile, active proteases in the urine limit the degradation of biomarkers, leading urinary proteomics with MS-based analysis to become one of the most attractive directions in disease biomarker discovery (94, 95). Most published literature utilized urine proteome as a target for detecting biomarkers to kidney and cardiovascular diseases, with only a few describing urine proteomics technologies on inflammatory arthritis (64, 96, 97). In research exploring urine biomarkers in four different arthritis [RA; PsA; osteoarthritis (OA); and inflammatory bowel diseases (IBD)], 50 most significant peptides, including 80% specific for one group only, and a minor overlap were found through urinary proteomics (98). However, the most detectable peptide markers in this study were collagen fragments previously derived from proteins functionally different from arthritis, which may be due to the filtration of the glomerulus or the limited uncovered nature of the peptides in the urine (98). The result indeed showed the potent application of urine proteomics and peptidomics in the future (99). More longitude cohort studies in a large number of samples should be carried out in the future.

## Conclusion

Over the past two decades, PsD is gradually considered a systematic inflammation that causes multiple associated comorbidities across the body rather than a simple disease cutaneous lesion (100). The emergence of skin presentation of psoriasis offers a unique opportunity for early management for those at high-risk systematic progression (101). Although existing reviews have already pointed out that imaging methods, such as ultrasound and MRI, can also become a valuable method to detect early the inflammatory lesions of joints, the expensive costs of exam fees and related equipment, and the long waiting time are limitations. Examination time and hard-interpreted imaging results for non-professional clinicians were all hurdles that hamper the prevalence of application on imaging examinations on patients with PsC (22, 25, 102). Consequently, a fast exam kit with an accessible kit becomes more necessary, suggesting an imperative need to explore a possible biomarker. The immense development and utilization in proteomics have provided an extraordinary chance to detail the molecular and mechanistic understanding of PsD pathways, decode the potential biomarkers, and investigate more effective intervention therapies (103, 104).

This review summarized the current approaches applied in the early PsA proteome. Compared with the traditional LC-MS/MS methods in proteogenomics, PEA provides more sensitive and specific detection for a more considerable range of low-abundant proteins in human blood and other body fluid samples (55–58). However, the need for the custom panel of biomarkers also restricted the exploration of the unknown proteins. Only a few studies that focused on psoriatic arthropathy finished their study with PEA technology. It highlighted the great need to perform high-throughput analyses in serum and tissues and other possible samples to discover PsA precursors. The future work on performing extensive integrative analysis will be undoubtedly challenging. Still, the increasing recognition of human proteome and consistent progression on proteomics technologies will become the most supportive foundation for challenging tasks.

## Author Contributions

YH: did the project administration, conceptualization, and methodology. FQ and YT: did the investigation and formal analysis. FQ, YT, AY, and XY: offered the resources. FQ wrote the original draft. YT: reviewed and edited the draft. YH: visualized the whole project and supervised the whole project. All authors contributed to the article and approved the submitted version.

## Funding

This project was funded by Beijing Municipal Science and Technology Project (Project) No. Z171100001017058 and National Natural Science Foundation of China No. 81773314.

## Conflict of Interest

The authors declare that the research was conducted in the absence of any commercial or financial relationships that could be construed as a potential conflict of interest.

## Publisher's Note

All claims expressed in this article are solely those of the authors and do not necessarily represent those of their affiliated organizations, or those of the publisher, the editors and the reviewers. Any product that may be evaluated in this article, or claim that may be made by its manufacturer, is not guaranteed or endorsed by the publisher.
